# Use of structured musculoskeletal examination routines in undergraduate medical education and postgraduate clinical practice – a UK survey

**DOI:** 10.1186/s12909-016-0799-6

**Published:** 2016-10-21

**Authors:** Kenneth F Baker, Sharmila Jandial, Ben Thompson, David Walker, Ken Taylor, Helen E Foster

**Affiliations:** 1 Musculoskeletal Research Group, NIHR Newcastle Biomedical Research Centre, Newcastle University and Newcastle upon Tyne Hospitals NHS Foundation Trust, NE2 4HH, Newcastle upon Tyne, UK; 2 Great North Children’s Hospital, Newcastle upon Tyne Hospitals NHS Foundation Trust, Newcastle upon Tyne, UK; 3 Policy, Ethics and Life Sciences Research Centre, Newcastle University, Newcastle upon Tyne, UK

**Keywords:** GALS, REMS, pGALS, Medical education, Musculoskeletal, Examination, Questionnaire, Undergraduate, Postgraduate, Clinical practice

## Abstract

**Background:**

Structured examination routines have been developed as educational resources for musculoskeletal clinical skills teaching, including Gait-Arms-Legs-Spine (GALS), Regional Examination of the Musculoskeletal System (REMS) and paediatric GALS (pGALS). In this study, we aimed to assess the awareness and use of these examination routines in undergraduate medical teaching in UK medical schools and UK postgraduate clinical practice.

**Methods:**

Electronic questionnaires were distributed to adult and paediatric musculoskeletal teaching leads at UK medical schools and current UK doctors in training.

**Results:**

Responses were received from 67 tutors representing teaching at 22/33 [67 %] of all UK medical schools, and 70 trainee doctors across a range of postgraduate training specialities. There was widespread adoption, at responding medical schools, of the adult examination routines within musculoskeletal teaching (GALS: 14/16 [88 %]; REMS: 12/16 [75 %]) and assessment (GALS: 13/16 [81 %]; REMS: 12/16 [75 %]). More trainees were aware of GALS (64/70 [91 %]) than REMS (14/67 [21 %]). Of the 39 trainees who used GALS in their clinical practice, 35/39 [90 %] reported that it had improved their confidence in musculoskeletal examination. Of the 17/22 responding medical schools that included paediatric musculoskeletal examination within their curricula, 15/17 [88 %] used the pGALS approach and this was included within student assessment at 4 medical schools.

**Conclusions:**

We demonstrate the widespread adoption of these examination routines in undergraduate education and significant uptake in postgraduate clinical practice. Further study is required to understand their impact upon clinical performance.

**Electronic supplementary material:**

The online version of this article (doi:10.1186/s12909-016-0799-6) contains supplementary material, which is available to authorized users.

## Background

Physical examination skills form an integral component of clinical practice and as such are recognised as essential learning outcomes in undergraduate medical education [1, 2]. Much attention has traditionally been focussed upon the four “core” bodily systems through the use of structured cardiovascular, respiratory, abdominal and neurological examination routines which are reinforced by assessment at qualification and postgraduate levels. In contrast, confidence and competence in musculoskeletal (MSK) examination is often poor and frequently overlooked in routine clinical practice [3, 4] despite the high prevalence of MSK symptoms in the general population [5].

The deficiency in MSK clinical skills education is arguably most apparent in the paediatric setting. Previous studies have demonstrated a significant lack of confidence in paediatric MSK examination skills within doctors from both primary and secondary care despite their previous exposure to adult MSK medicine [6, 7]. Furthermore, the unique spectrum of paediatric MSK presentations and their relationship to normal development, the challenges of history taking from the young child / carer dyad and the acquisition of paediatric MSK clinical skills are all different to those relevant in the adult setting [8].

To help address the unmet need for improved education in both adult and paediatric MSK clinical skills, a series of structured examination routines have been developed including the Gait-Arms-Legs-Spine approach (GALS – 1992) [9], the Regional Examination of the Musculoskeletal System (REMS – 2004) [10] and most recently paediatric GALS (pGALS – 2006) [8] and paediatric REMS (pREMS – 2011) [11]. For GALS, REMS and pGALS, educational resources have been made freely available to medical schools via booklets, DVDs and online with the charitable support of Arthritis Research UK [12]. Resources for pREMS were not available at the time of this study but video demonstrations are now available on the Paediatric Musculoskeletal Matters website [13] with signposting to this via the Arthritis Research UK website.

In this study, we aimed to assess the impact of GALS, REMS and pGALS in terms of their use within the teaching and assessment of MSK clinical skills at UK medical schools. A further aim was to understand the impact of these tools in clinical practice by UK trainee doctors.

## Methods

Questionnaires (see Additional files 1, 2 and 3) were designed to assess awareness, use and clinical relevance of the GALS, REMS and pGALS examination routines and their inclusion in undergraduate assessments through a combination of multiple choice questions, 5-point Likert statements and free-text responses. Questionnaires were pretested and piloted by the study authors and an additional four researchers to assess face and construct validity. Suggestions for improved clarity of the layout and question wording were incorporated within the design of the survey before distribution. Electronic questionnaires were distributed in 2013 by an invitation email containing a link to the survey website, where respondents could complete the survey anonymously. Three main respondent groups were targeted, namely: (i) adult MSK tutors, (ii) those responsible for delivering paediatric MSK teaching (hereafter referred to as “paediatric MSK tutors”) and (iii) current trainee doctors. Adult MSK tutors comprised the lead tutor of adult MSK medicine at each of the 33 UK medical schools as identified by a contact list updated by Arthritis Research UK. In contrast, there was no available contact list for paediatric MSK tutors. For the purposes of this study, our pragmatic definition of paediatric MSK tutors included both locally identified child health teaching leads at UK medical schools, plus UK paediatric rheumatology consultants and specialist registrars. We were able to identify and distribute questionnaires to 20 child health teaching leads, 15 UK paediatric rheumatology specialist registrars and 27 UK paediatric rheumatology consultants.

There were 54,055 doctors in training in the UK in 2013 [14], and distributing surveys directly to this group proved logistically challenging due to the lack of an accessible central trainee database and their wide geographical distribution across multiple training bodies. Engagement with current trainee doctors who graduated from a UK medical school was therefore achieved via an online medical community (www.doctors.net.uk) with a gift voucher prize draw offered as an incentive to participation. This survey was made available to 12,000 unselected UK doctors via an educational bulletin email. The questionnaire was viewed by approximately 2,400 (20 %) recipients with 70 eligible responses from current UK trainee doctors received. Responses for the REMS and pGALS section of the questionnaire were not received from 3 trainees. An additional 11 responses were received from doctors who were either not based in the UK and/or not currently in a clinical training post and were therefore excluded from our analysis.

## Results

### Survey respondents

Responses were received from 19 adult MSK tutors at 16 medical schools, and 48 paediatric MSK tutors at 20 medical schools. Combined, the tutor survey responses represented teaching at a total of 22/33 (67 %) of all UK medical schools: 14 England, 5 Scotland, 2 Wales and 1 Northern Ireland. Responses were received from 70 trainees in a range of different specialities including the UK Foundation Programme, primary care and various secondary care specialities (Table 1).

**Table 1:** Demographics of trainee doctors who responded to the survey

Demographic	Value
Number of eligible responses received	70
Female: n (%) respondents	42 (60 %)
Age: median (IQR) years	29 (27–31)
Time since primary medical qualification: median (IQR) years	4 (2–5)
Current training speciality: n (%)	
General Practice	20 (29 %)
UK Foundation Programme	18 (26 %)
Paediatrics	5 (7 %)
Acute Care Common Stem (ACCS)	5 (7 %)
Core Medical Training (CMT)	4 (6 %)
Orthopaedics	3 (4 %)
Emergency Medicine	3 (4 %)
Core Surgical Training	2 (3 %)
Other specialities	10 (14 %)

### Tutor awareness of MSK examination routines and use in undergraduate teaching

**Fig. 1:**
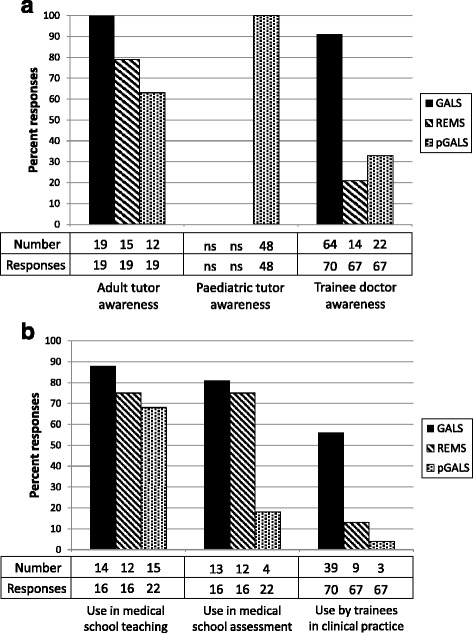
Awareness (**a**) and use (**b**) of structured musculoskeletal examination routines by undergraduate tutors and postgraduate trainee doctors. Tutor awareness is shown as percentage of adult tutor responses received for GALS and REMS, and as percentage of total tutor responses for pGALS. Use in medical school teaching and assessment is shown as percentage of medical schools at which responding tutors are based. ns: not surveyed

Tutor awareness of the adult MSK examination routines was high: GALS: 19/19 [100 %], REMS: 15/19 [79 %] of adult MSK tutor responses (Fig. 1). This awareness translated into impact as responses showed that GALS and REMS routines were incorporated within the undergraduate teaching programmes of 14/16 (88 %) and 12/16 (75 %) of responding medical schools respectively. These examination routines were incorporated within the formal assessment of medical students (GALS: 13/16 [81 %], REMS: 12/16 [75 %] responding medical schools). The format of these adult MSK assessments was not requested in the survey.

Based upon the 67 tutor responses, paediatric MSK examination has been included within general MSK and/or paediatric teaching programmes at 17/22 (77 %) medical schools. Commonly cited reasons for not including paediatric MSK examination within teaching programmes included time constraints and a lack of tutors who were confident in delivering paediatric MSK teaching locally.

All 48 paediatric tutors surveyed were aware of pGALS, whilst awareness was limited to 12/19 (63 %) of responding adult MSK tutors. Of the 17 responding medical schools that included paediatric MSK examination within their teaching programmes, 15/17 (88 %) taught the pGALS approach and 13/17 (76 %) allowed for practice of examination technique with children with a spectrum of MSK problems. Where pGALS was taught, the examination routine was incorporated within the formal assessment of students in 4/15 (27 %) of these medical schools as part of an objective structured clinical examination (OSCE).

### Trainee doctor awareness of MSK examination routines and reported use in clinical practice

Awareness of GALS was high amongst trainees (64/70 [91 %] responses), though less were aware of REMS (14/67 [21 %] responses). Concomitantly, more trainees reported using GALS (39/70 [56 %] responses) than REMS (9/67 [13 %] responses) in their current clinical practice (Fig. 1). Of the 39 trainees who used GALS, 35/39 (90 %) felt it had improved their ability to detect significant MSK pathology.

Undergraduate exposure to paediatric MSK medicine appeared to be low in the respondents; only 15/67 (22 %) of trainees recalled any form of teaching in paediatric MSK examination at medical school. Nevertheless, 22/67 (33 %) of trainees across a broad range of specialities were aware of the pGALS, suggesting some postgraduate exposure to this examination routine. There was a wide variation in the degree of exposure to paediatric medicine within the different trainee speciality groups; 32/70 (46 %) of trainees reported clinical contact with children on at least a weekly basis. Of these trainees, 3/32 (9 %) use pGALS in their current clinical practice, all of whom were general paediatric trainees.

### Perceived quality of MSK examination routines

The majority of tutors and trainees alike rated the quality of the examination routines highly in Likert statement responses. Positive free-text responses focussed especially around the simplicity and ease of remembering the sequence of the examination routines. However, the GALS routine was identified as over-simplistic for use in clinical practice by several tutors and trainees. Furthermore, several paediatric tutors remarked that the pGALS approach can encourage a superficial rote-learning of paediatric MSK examination rather than a detailed understanding; however, the influence of the local curriculum and student factors in this context was not discussed.

## Discussion

In this survey we examined the use of structured approaches to MSK examination (GALS, REMS and pGALS) within undergraduate teaching at UK medical schools, and then explored the awareness and use of these routines by training-grade doctors in their current clinical practice. Our data shows that GALS is most widely taught, most likely to feature in undergraduate assessments, most likely to be used in clinical practice and leads to improved self-rated confidence in MSK examination by the trainee doctors who responded to our survey. Our results thus provide encouraging evidence to support the acceptance of adult MSK examination as a core skill in UK undergraduate medical education and a proficiency which is relevant and useful to trainees in their future clinical practice.

With regard to adult general MSK examination, and using Miller’s pyramid approach to clinical competence [15], we present evidence to support the widespread knowledge of GALS (“knows how”) and incorporation of this within undergraduate teaching and assessment (“shows how”). However, the successful mastery of GALS in clinical practice, represented by the apical “does” level of Miller’s pyramid, remains difficult to assess. Previous studies include clinical notes audit [16] and surveys of orthopaedic and rheumatology clinicians [17], and these have suggested a low use of GALS in clinical practice. It is therefore particularly striking to observe that the majority of trainees in our study report use of GALS in their current clinical practice and that GALS has improved their self-confidence in adult MSK examination, an effect of the examination routine which has also been observed previously [18, 19].

There is an apparent discordance between the extensive integration of the REMS examination routine within medical student teaching programmes and relatively low levels of awareness amongst current trainee doctors. Although we cannot confirm, it is plausible that our respondents may adopt a regional examination approach but not refer to this as ‘REMS’. Alternatively the conscious use of the REMS routine may be more applicable to MSK specialities such as rheumatology and orthopaedics than more general areas. This finding highlights the limited nature of survey-based research and indicates that more in-depth study is required to better understand the apparent underuse of the REMS routine in clinical practice.

The incorporation of pGALS within educational assessments at many medical schools is an important observation given the evidence to support that “assessment drives learning” [20, 21]. A previous survey of UK medical schools by Jandial et al. (2009) [22] provided evidence of paediatric MSK clinical skills teaching at 9/23 (39 %) of responding medical schools, which has substantially increased to 17/22 (77 %) in this current study. Furthermore, it is noteworthy that the majority of medical schools in our current survey provide opportunities to practice MSK examination skills with children with MSK disorders, in contrast to the largely lecture-based teaching previously identified [22]. While it is noted that many trainees were unable to recall any undergraduate tuition in paediatric MSK examination, this is likely to reflect the evolution of paediatric MSK teaching in medical schools since the relatively recent emergence of pGALS (2006). These observations suggest a need to further embed MSK clinical skills within child health teaching at undergraduate level, and the need to learn skills in appropriate clinical contexts in order to aid understanding and avoid low-level learning by rote.

Notable strengths of this study include the wide geographical survey of educational practice at a national level across the United Kingdom and the inclusion of responses from trainee doctors across a broad range of postgraduate specialities. Nevertheless, there are several limitations to this study. First, tutors at only two thirds of UK medical schools responded, leading to a concern that non-response was biased towards those schools with low awareness and use of MSK resources. Second, a contact list of paediatric MSK tutors was (and remains) unavailable, and thus we distributed surveys to those who we identified as likely being responsible for delivering undergraduate paediatric MSK teaching, namely general child health teaching leads, and consultants and specialist registrars in paediatric rheumatology. However, despite our best efforts, we were unable to contact the child health teaching lead at 13 medical schools. It is further possible that additional tutors deliver paediatric MSK teaching but did not fall within our targeted survey groups. The mixed strategy for targeting paediatric MSK tutors that we adopted resulted in a greater number of responses versus the adult MSK tutor group (48 vs. 19 respectively). It is thus possible that our survey may underrepresent the use of the adult MSK resources – however, the universally high reported use of GALS would suggest this is likely to have had little effect upon our results. Distributing surveys to trainee doctors proved challenging owing to the fluid and geographically disperse nature of this group. Through the use of an online community we were able to survey across a wide range of trainee doctor specialities, although the response rate was disappointingly low (only 0.5 % of the total number of doctors who received the survey invitation). A response bias may have affected our result – i.e. responding trainee doctors may differ from non-responders in their exposure to and enthusiasm for MSK medicine – and it is thus impossible to extrapolate the results of our survey to the wider UK trainee doctor population. Furthermore, the self-reported use of and confidence in MSK examination routines by trainee doctors may not necessarily equate to competent performance in clinical practice, although the assessment of this lies outside of the scope of a questionnaire-based survey.

## Conclusions

In summary, we demonstrate the widespread adoption of GALS, REMS and pGALS in the teaching of MSK clinical skills at UK medical schools, with evidence of uptake and use in postgraduate clinical practice. The impact of these examination routines upon medical student teaching will be affected by wider factors including the context and emphasis placed upon MSK skills teaching within the local curriculum, the resources that are available to deliver such teaching and the methods of assessment used. Further work to scope the delivery of and barriers to MSK teaching, particularly in the paediatric setting, would be beneficial. The ultimate affirmation of the value of structured MSK examination routines would be to demonstrate a positive effect upon clinical performance, and this would require a different methodological approach to that described here. Further qualitative, observational as well as quantitative research is required to improve our understanding of the impact of these structured routines upon the mastery of MSK examination skills in real-life clinical settings and the consequent delivery of patient benefit.

## Additional files

Additional file 1: Lead Adult Musculoskeletal Tutor Questionnaire. (DOC 54 kb)
Additional file 2: Paediatric Tutor Questionnaire. (DOCX 21 kb)
Additional file 3: Trainee Doctor Questionnaire. (DOC 51 kb)

## References

[1] General Medical Counci (2009). Tomorrow’s doctors.

[2] World Federation for Medical Education (2012). Basic medical education WFME Global Standards for Quality Improvement.

[3] Dequeker J, Esselens G, Westhovens R (2007). Educational issues in rheumatology. The musculoskeletal examination: a neglected skill. Clin Rheumatol.

[4] Sirisena D, Begum H, Selvarajah M, Chakravarty K (2011). Musculoskeletal examination--an ignored aspect. Why are we still failing the patients?. Clin Rheumatol.

[5] Jordan KP, Kadam UT, Hayward R, Porcheret M, Young C, Croft P (2010). Annual consultation prevalence of regional musculoskeletal problems in primary care: an observational study. BMC Musculoskelet Disord.

[6] Myers A, McDonagh JE, Gupta K (2004). More ‘cries from the joints’: assessment of the musculoskeletal system is poorly documented in routine paediatric clerking. Rheumatology (Oxford).

[7] Jandial S, Myers A, Wise E, Foster HE (2009). Doctors likely to encounter children with musculoskeletal complaints have low confidence in their clinical skills. J Pediatr.

[8] Foster HE, Kay LJ, Friswell M, Coady D, Myers A (2006). Musculoskeletal screening examination (pGALS) for school-age children based on the adult GALS screen. Arthritis Rheum.

[9] Doherty M, Dacre J, Dieppe P, Snaith M (1992). The ‘GALS’ locomotor screen. Ann Rheum Dis.

[10] Coady D, Walker D, Kay L (2004). Regional Examination of the Musculoskeletal System (REMS): a core set of clinical skills for medical students. Rheumatology (Oxford).

[11] Foster H, Kay L, May C, Rapley T (2011). Pediatric regional examination of the musculoskeletal system: a practice- and consensus-based approach. Arthritis Care Res (Hoboken).

[12] Arthritis Research UK website. http://www.arthritisresearchuk.org. Accessed 5 July 2016.

[13] Paediatric Musculoskeletal Matters. http://www.pmmonline.org. Accessed 5 July 2016.

[14] General Medical Council. National Training Survey 2013 Report. General Medical Council 2013. http://www.gmc-uk.org/National_training_survey_2013_key_findings_report_1013.pdf_56436856.pdf. Accessed 24 Aug 2016

[15] Miller GE (1990). The assessment of clinical skills/competence/performance. Acad Med.

[16] Marshall RW, Hull RG (2004). For crying out loud: musculoskeletal assessment of inpatients referred to rheumatology. Rheumatology (Oxford).

[17] Blake T (2014). Teaching musculoskeletal examination skills to UK medical students: a comparative survey of Rheumatology and Orthopaedic education practice. BMC Med Educ.

[18] Lillicrap MS, Byrne E, Speed CA (2003). Musculoskeletal assessment of general medical in-patients--joints still crying out for attention. Rheumatology (Oxford).

[19] Fox RA, Dacre JE, Clark CL, Scotland AD (2000). Impact on medical students of incorporating GALS screen teaching into the medical school curriculum. Ann Rheum Dis.

[20] Raupach T, Brown J, Anders S, Hasenfuss G, Harendza S (2013). Summative assessments are more powerful drivers of student learning than resource intensive teaching formats. BMC Med.

[21] Wormald BW, Schoeman S, Somasunderam A, Penn M (2009). Assessment drives learning: an unavoidable truth?. Anat Sci Educ.

[22] Jandial S, Rapley T, Foster H (2009). Current teaching of paediatric musculoskeletal medicine within UK medical schools--a need for change. Rheumatology (Oxford).

