# Supplementary material for: Use of structured musculoskeletal examination routines in undergraduate medical education and postgraduate clinical practice – a UK survey
Source: BMC Med Educ. 2016 Oct 21;16:277. doi: 10.1186/s12909-016-0799-6 (PMC5073898; doi:10.1186/s12909-016-0799-6)
Supplement: Additional file 1: — Lead Adult Musculoskeletal Tutor Questionnaire. (DOC 54 kb) [file 12909_2016_799_MOESM1_ESM.doc]

Lead Adult Musculoskeletal Tutor Questionnaire

Introduction

Thank you for taking the time to complete this short survey. All of the responses you provide are collected and processed anonymously.

Please note that it is not possible to navigate backwards to change your answers once submitted.

Click on the next button below to begin.

Please could you tell us the medical school in which you provide medical student teaching? If your medical school is not listed or if you teach at multiple medical schools, please select the "other" option and provide further details in the box below.

Please note this question is solely to ensure that we have responses from an adequate geographical coverage of the UK. The presentation of data from this survey will not link your responses to your host medical school.

**SELECT FROM DROPDOWN LIST OF 33 UK MEDICAL SCHOOLS OR FREE TEXT**

Booklet

This section of the survey focusses on the Arthritis Research UK educational booklet

Are you aware of the Arthritis Research UK (ARUK) “Clinical Assessment of the Musculoskeletal System” booklet for medical students and health professionals? **YES/NO**

*If NO, skip to next section*

*If YES*: Is the Arthritis Research UK booklet used in the undergraduate teaching of musculoskeletal history and examination at your medical school? **YES/NO**

*If YES*: How is the booklet used? (select as many as apply)

1. distributed to students as educational resource to complement teaching (i.e. as “further reading”)
2. in its entirety as a framework for teaching programme (i.e. a “gold standard”)
3. only selected parts of booklet are incorporated within teaching programme (if this selected then please specify which parts in the comments field below) **Free text**
4. other (please specify in the comments field below) – **free text**

*If NO:* Why is the booklet not used? (select as many as apply)

1. lack of availability of handbook for distribution to students
2. an alternative handbook/educational resource is already in use within the medical school
3. I disagree with the format or content of the handbook
4. Other – **free text**

How would you rate your experience of the Arthritis Research UK booklet? Please select your preferred responses to the items listed below. *(5 point Likert scale: strongly agree, agree, neutral, disagree, strongly disagree)*

1. The structure of booklet is well-organised
2. The diagrams within the booklet help to explain underlying concepts
3. There is sufficient detail on history-taking skills
4. The key important points are sufficiently emphasised.
5. The terminology used is appropriate for a medical student audience
6. The photographs of examination technique are clear to follow
7. The written instructions for examination technique are easy to follow
8. There is sufficient detail on investigations
9. There is enough text
10. There are enough diagrams
11. The DVD is a useful addition to the booklet

For the intended clinical undergraduate medical student audience, the booklet is

1. lacking enough detail, aimed at too low a level
2. sufficiently detailed, aimed at an appropriate level
3. overly detailed, aimed at too high a level

What do you like about the ARUK booklet? (optional) **Free text**

What do you dislike about the ARUK booklet? Could it be improved in any way? (optional) **Free text**

GALS

This section of the survey focusses on the Gait-Arms-Legs-Spine (GALS) approach to musculoskeletal examination.

Are you aware of the GALS (Gait-Arms-Legs-Spine) approach to musculoskeletal screening examination? **YES/NO**

*If NO, skip to next section*

*If YES:* Do you teach the GALS approach as part of your course? **YES/NO**

*If NO*: What alternative approach to musculoskeletal screening examination do you teach and why? **Free text**

*If YES:* Are students formally assessed in their ability to perform a GALS screening examination during/after the course? **YES/NO/UNSURE**

How would you rate your experience of the GALS approach? Please select your preferred responses to the items listed below. *(5 point Likert scale: strongly agree, agree, neutral, disagree, strongly disagree)*

1. It is easy to remember
2. It comprehensively covers the full range of musculoskeletal examination expected of a non-specialist
3. It has sufficient detail in examination technique
4. It is sufficiently concise for routine use
5. It is sufficiently detailed to detect the vast majority of musculoskeletal pathologies

What do you like about the GALS approach? (optional) **Free text**

What do you dislike about the GALS approach? Could it be improved in any way? (optional) **Free text**

REMS

This section of the survey focusses on your experience of the various different Regional Examination of the Musculoskeletal System (REMS) examination routines, as detailed in the Arthritis Research UK "Clinical Assessment of the Musculoskeletal System" booklet.

When teaching medical students, do you distinguish between a general screening musculoskeletal examination versus detailed individual joint examination routines? **YES / NO**

Are you aware of the REMS (Regional Examination of the Musculoskeletal System) examination routines? **YES/NO**

*If NO, skip to next section*

*If YES:* Do you teach the regional musculoskeletal examination routines as detailed in the Arthritis Research UK handbook in your medical school? **YES/NO**

*If NO:* What alternative approach to regional musculoskeletal examination do you use and why? **Free text**

*If YES:* Does the REMS approach feature as part of the formal assessment of regional musculoskeletal examination for medical students in your medical school? **YES/NO/UNSURE**

How would you rate your experience of the REMS approach? Please select your preferred responses to the items listed below. *(5 point Likert scale: strongly agree, agree, neutral, disagree, strongly disagree)*

1. The examination routines are easy to remember
2. They comprehensively cover the full range of musculoskeletal examination expected of a non-specialist
3. The examination routines are sufficiently concise for routine use
4. The examination routines are sufficiently detailed to detect the vast majority of musculoskeletal pathologies

What do you like about the REMS examination routines? (optional) **Free text**

What do you dislike about the REMS approach? Can you suggest any ways in which this approach could be improved? (optional) **Free text**

pGALS

This section of the survey focusses on your experience (if any) of using the Paediatric Gait-Arms-Legs-Spine (pGALS) approach to musculoskeletal examination in school-aged children.

[1]

Does the undergraduate musculoskeletal teaching course at your medical school include paediatric musculoskeletal examination? **YES/NO/UNSURE**

*If NO*: Why is paediatric musculoskeletal examination not included as part of your undergraduate musculoskeletal course? (select as many as apply)

1. covered elsewhere in undergraduate curriculum
2. insufficient time in teaching programme
3. there are more important topics to cover than paediatric musculoskeletal examination
4. paediatric musculoskeletal examination is too specialised and hence will unlikely be of use to students in their future medical careers
5. lack of teachers in the department who are confident in teaching paediatric musculoskeletal examination
6. other (please specify below) – **Free text**

[2]

Are you aware of the pGALS (paediatric Gait-Arms-Legs-Spine) approach to musculoskeletal examination in school-aged children?

**YES/NO**

*If NO, then skip to end of questionnaire*

Are you aware of the Arthritis Research UK educational video for pGALS examination?

**YES/NO**

*If YES*: Have you watched the Arthritis Research UK educational video for pGALS examination?

**YES/NO**

*If YES*: How have you viewed the ARUK pGALS video? (please select as many as apply)

1. DVD provided by Arthritis Research UK
2. Online video via Arthritis Research UK website
3. Online video via YouTube
4. Other (please specify) – **Free text**

Do you teach the pGALS approach as part of your musculoskeletal course? **YES/NO/UNSURE**

*If NO or UNSURE,* What alternative approach to paediatric musculoskeletal examination do you teach and why? **Free text** *then skip to [3]*

Which of the following most closely matches the way in which you teach pGALS within your musculoskeletal course?

1. pGALS checklist or link to pGALS internet resources provided to students as “further reading” for those who are interested
2. Students watched ARUK video only
3. Students watched ARUK video with accompanying lecture
4. Students given lecture on pGALS approach without being shown ARUK video
5. Other (please specify below) – **Free text**

Do you provide your students with (or inform them of) any of the following pGALS learning resources? (select as many as apply)

1. ARUK pGALS DVD
2. Link to pGALS section of ARUK website
3. Link to ARUK pGALS video on YouTube
4. pGALS article featured in the ARUK “Hands-On” publication (June 2008, No. 15)
5. pGALS section of a textbook – if so, please specify which textbook in the comments box below. **Free text**
6. other (please specify in the comments box below) – **Free text**

Do students have the opportunity to practice pGALS examination as part of your musculoskeletal course? (please select as many as apply)

1. no opportunity
2. practice pGALS examination routine on each other
3. practice pGALS examination routine on healthy children
4. practice pGALS examination routine on children with musculoskeletal disorders

Are students assessed in their ability to perform a pGALS assessment of a child during/after your musculoskeletal course? **YES/NO**

*If YES*: How are students assessed in their ability to perform a pGALS assessment during/after your musculoskeletal course? (please select as many as apply)

1. assessment/feedback from tutors on examination technique during pGALS teaching session
2. written/theoretical exercises (e.g. clinical cases, problem-solving)
3. as part of an OSCE (Objective Structured Clinical Examination)
4. other (please specify below)– **Free text**

*If YES*: Is ability to perform a pGALS examination a defined learning outcome for your musculoskeletal course? (select one)

1. not a learning outcome
2. a desirable learning outcome (i.e. encouraged but not essential to “pass” the course)
3. a mandatory learning outcome (i.e. required to “pass” the course)

[3]

How would you rate your experience of the pGALS approach? Please select your preferred responses to the items listed below. *(5 point Likert scale: strongly agree, agree, neutral, disagree, strongly disagree)*

1. It is easy to remember
2. It comprehensively covers the full range of paediatric musculoskeletal examination expected of a non-specialist
3. It covers sufficient detail in examination technique
4. It is sufficiently concise for routine use
5. It has sufficient scope to detect the vast majority of significant joint abnormalities in paediatric musculoskeletal medicine

What do you like about the pGALS approach? (optional) **Free text**

What do you dislike about the pGALS approach? Can you suggest any ways in which this approach could be improved? (optional) **Free text**

Have you encountered any difficulties in teaching pGALS within an undergraduate medical school musculoskeletal course? If so, what have these been? (optional) **Free text**

End of Questionnaire

Thank you for taking the time to complete this survey your - responses have provided us with invaluable information.

We would be interested in hearing further views from some of our respondents. If you would be interested in participating in a short structured telephone interview at a later date, please indicate this by entering your email address in the box below. **Free text**
