# Supplementary material for: Use of structured musculoskeletal examination routines in undergraduate medical education and postgraduate clinical practice – a UK survey
Source: BMC Med Educ. 2016 Oct 21;16:277. doi: 10.1186/s12909-016-0799-6 (PMC5073898; doi:10.1186/s12909-016-0799-6)
Supplement: Additional file 2: — Paediatric Tutor Questionnaire. (DOCX 21 kb) [file 12909_2016_799_MOESM2_ESM.docx]

Paediatric Tutor Questionnaire

Thank you for taking the time to complete this short survey. All of the responses you provide are collected and processed anonymously.

Please note that it is not possible to navigate backwards to change your answers once submitted.

Click on the next button below to begin.

Please could you tell us the medical school in which you provide medical student teaching? If your medical school is not listed, please select the "other" option and provide further details in the box below. If you provide teaching at multiple medical schools, please select the one at which you provide the majority of your teaching.

Please note this question is solely to ensure that we have responses from an adequate geographical coverage of the UK. The presentation of data from this survey will be anonymous and will not link your responses to your host medical school.

**Select from dropdown list of 33 UK medical schools or free text**

[1]

Are you aware of the pGALS (paediatric Gait-Arms-Legs-Spine) approach to musculoskeletal examination in school-aged children?

**YES/NO**

*If YES then skip to [2]*

*If NO, then complete remainder of [1] then skip to end of questionnaire*

Does the undergraduate musculoskeletal teaching course at your medical school include paediatric musculoskeletal examination? **YES/NO/UNSURE**

*If NO*: Why is paediatric musculoskeletal examination not included as part of your undergraduate musculoskeletal course? (please select as many as apply)

1. covered elsewhere in undergraduate curriculum (if so, where? – please specify in text box below)
2. insufficient time in teaching programme
3. there are more important topics to cover than paediatric musculoskeletal examination
4. paediatric musculoskeletal examination is too specialised and hence will unlikely be of use to students in their future medical careers
5. lack of teachers in the department who are confident in teaching paediatric musculoskeletal examination
6. Unsure – I am not involved in setting the course curriculum
7. other (please specify below) – **Free text**

What approach to paediatric musculoskeletal teaching do you teach and why? Is there a specific reason why you do not teach the pGALS approach? **Free text** *then skip to end of questionnaire*

[2]

Are you aware of the Arthritis Research UK educational video for pGALS examination?

**YES/NO**

*If YES*: Have you watched the Arthritis Research UK educational video for pGALS examination?

**YES/NO**

*If YES*: How have you viewed the Arthritis Research UK pGALS video? (please select as many as apply)

1. DVD provided by Arthritis Research UK
2. Online video via Arthritis Research UK website
3. Online video via YouTube
4. Other (please specify) – **Free text**

Does the undergraduate paediatric teaching course at your medical school include paediatric musculoskeletal examination? **YES/NO/UNSURE**

*If NO*: Why is paediatric musculoskeletal examination not included as part of the undergraduate paediatric course at your medical school? (please select as many as apply)

1. covered elsewhere in undergraduate curriculum (if so, where? – please specify in text box below)
2. insufficient time in teaching programme
3. there are more important topics to cover than paediatric musculoskeletal examination
4. paediatric musculoskeletal examination is too specialised and hence will unlikely be of use to students in their future medical careers
5. lack of teachers in the department who are confident in teaching paediatric musculoskeletal examination
6. Unsure – I am not involved in setting the course curriculum
7. other (please specify below) – **Free text**

Is the pGALS approach taught as part of the undergraduate paediatric course at your medical school? **YES/NO/UNSURE**

*If NO:* What alternative approach to paediatric musculoskeletal examination do you teach and why? **Free text** *then skip to [3]*

Which of the following most closely matches the way in which you teach pGALS within your musculoskeletal course?

1. pGALS checklist or link to pGALS internet resources provided to students as “further reading” for those who are interested
2. Students watched Arthritis Research UK pGALS video only
3. Students watched Arthritis Research UK pGALS video with accompanying lecture
4. Students given lecture on pGALS approach without being shown Arthritis Research UK video
5. Other (please specify below) – **Free text**

Do you provide your students with any of the following pGALS learning resources? (please select as many as apply)

1. Arthritis Research UK pGALS DVD
2. Link to pGALS section of Arthritis Research UK website
3. Link to Arthritis Research UK pGALS video on YouTube
4. pGALS article featured in the Arthritis Research UK “Hands-On” publication (June 2008, No. 15)
5. pGALS section of a textbook – if so, please specify which textbook in the comments box below. **Free text**
6. other (please specify in the comments box below) – **Free text**

Do students have the opportunity to practice pGALS examination as part of your musculoskeletal course? (please select as many as apply)

1. no opportunity
2. practice pGALS examination routine on each other
3. practice pGALS examination routine on healthy children
4. practice pGALS examination routine on children with musculoskeletal disorders
5. Other (please specify below) – **Free text**

Are students assessed in their ability to perform a pGALS assessment of a child during/after the undergraduate paediatric course at your medical school? **YES/NO/UNSURE**

*If YES*: How are students assessed in their ability to perform a pGALS assessment of a child during/after the undergraduate paediatric course? (please select as many as apply)

1. assessment/feedback from tutors on examination technique during pGALS teaching session
2. written/theoretical exercises (e.g. clinical cases, problem-solving)
3. as part of an OSCE (Objective Structured Clinical Examination)
4. other (please specify below)– **Free text**

*If YES*: Is ability to perform a pGALS examination a defined learning outcome for your musculoskeletal course? (select one)

1. not a learning outcome
2. a desirable learning outcome (i.e. encouraged but not essential to “pass” the course)
3. a mandatory learning outcome (i.e. required to “pass” the course)

[3]

How would you rate your experience of the pGALS approach? Please select your preferred responses to the items listed below. *(5 point Likert scale: strongly agree, agree, neutral, disagree, strongly disagree)*

1. It is easy to remember
2. It comprehensively covers the full range of paediatric musculoskeletal examination expected of a non-specialist
3. It covers sufficient detail in examination technique
4. It is sufficiently concise for routine use
5. It has sufficient scope to detect the majority of significant joint abnormalities in paediatric musculoskeletal medicine

What do you like about the pGALS approach? (optional) **Free text**

What do you dislike about the pGALS approach? Can you suggest any ways to improve this approach? (optional) **Free text**

Have you encountered any difficulties in teaching pGALS within an undergraduate medical school paediatric course? If so, what have these been? (optional) **Free text**

Are there any further educational resources that you require to facilitate the teaching of pGALS? **Free text**

Do you have any further comments? **Free text**

End of Questionnaire

Thank you for taking the time to complete this survey your - responses have provided us with invaluable information.

We would be interested in hearing further views from some of our respondents. If you would be interested in participating in a short structured telephone interview at a later date, please indicate this by entering your email address in the box below. **Free text**
