# Supplementary material for: Use of structured musculoskeletal examination routines in undergraduate medical education and postgraduate clinical practice – a UK survey
Source: BMC Med Educ. 2016 Oct 21;16:277. doi: 10.1186/s12909-016-0799-6 (PMC5073898; doi:10.1186/s12909-016-0799-6)
Supplement: Additional file 3: — Trainee Doctor Questionnaire. (DOC 51 kb) [file 12909_2016_799_MOESM3_ESM.doc]

Trainee Doctor Questionnaire

Thank you for taking the time to complete this short survey. All of the responses you provide are collected and processed anonymously.

Please note that it is not possible to navigate backwards to change your answers once submitted.

Click on the next button below to begin.

Eligibility questions

Did you undertake your medical training at a UK medical school? **YES/NO**

*If NO, then skip to end of questionnaire*

Are you currently working as a doctor within the United Kingdom? **YES/NO**

*If NO, then skip to end of questionnaire*

Does your current job role involve any element of face-to-face clinical contact with patients? (i.e. history-taking and/or clinical examination) **YES/NO**

*If NO, then skip to end of questionnaire*

Tell us about yourself

Which of the following best describes your current job role?

1. Trainee doctor (i.e. Foundation, Core, Speciality, Academic or LAT trainee)
2. Trainee doctor currently on an out-of-programme
3. (OOP) placement
4. Junior or middle-grade doctor not currently in training (e.g. Trust-grade, LAS, locum posts)
5. General practitioner (i.e. completed GP training)
6. Consultant (if so, please specify which speciality below)

*Note – if respondent answers d), e), or f) then their responses are not eligible for analysis*

What is your CURRENT training programme speciality? (for dual-accrediting

trainees, please select your primary speciality)

1. Core Medical Training
2. Core Surgical Training
3. Foundation Programme (F1, F2)
4. General Practice
5. Orthopaedics
6. Paediatrics
7. Rheumatology
8. Other – (please specify below) **Free text**

Please select the medical school at which you completed your clinical training. **List of 33 UK medical schools, with “Other” and free text option**

At what level did you enter medical school?

1. Undergraduate (including intercalated BSc/BA/BMedSci etc.)
2. Graduate

In what year did you graduate with your final clinical medical degree?

1. 2012
2. 2011
3. 2010
4. 2009
5. 2008
6. 2007
7. 2006
8. 2005
9. Other (please specify below) **Free text**

Please could you tell us your age?

1. I would prefer not to say
2. 20
3. 21
4. 22
5. 23
6. 24

(etc. up to 70)

Please could you select your gender?

1. Male
2. Female
3. I would prefer not to say

Confidence in Adult Musculoskeletal Examination

With regards to ADULT patients, how confident are you in your ability to RECOGNISE the following significant musculoskeletal pathologies and refer for further specialist management where appropriate? (please rate your confidence in recognition only, and not in subsequent management) *(10 point scale from 10 = very confident to 1 = no confidence)*

1. hip fracture
2. ankylosing spondylitis
3. prolapsed intervertebral disc causing spinal cord or cauda equina compression
4. osteoarthritis
5. systemic lupus erythematosus
6. rheumatoid arthritis
7. septic arthritis

Confidence in Paediatric Musculoskeletal Examination

In your CURRENT post, roughly how frequently would you provide care for school-aged children?

1. daily
2. weekly
3. monthly
4. yearly
5. never

With regards to SCHOOL-AGED CHILDREN, how confident are you in your ability to diagnose the following significant musculoskeletal pathologies and refer for further specialist management where appropriate? (please rate your confidence in recognition only, and not in subsequent management. Please provide answers even if you do not regularly provide care for children) *(10 point visual scale from 10 = very confident to 1 = no confidence)*

1. Osgood-Schlatter syndrome
2. greenstick fracture of radius
3. Septic arthritis
4. Perthes disease
5. Slipped upper femoral epiphysis
6. Polyarticular JIA (juvenile idiopathic arthritis)

Arthritis Research UK Booklet

Are you aware of the Arthritis Research UK (ARUK) “Clinical Assessment of the Musculoskeletal System” booklet for medical students and health professionals? **YES/NO**

*If NO – skip to next section*

How did you become aware of the ARUK booklet?

1. Given to me as a student at medical school
2. Via ARUK website
3. Word-of-mouth
4. Other (please specify) **Free text**

GALS (Gait-Arms-Legs-Spine) Approach

Are you aware of the GALS approach (Gait-Arms-Legs-Spine) for musculoskeletal

examination of adult patients? **YES/NO**

*If NO*, *skip to next section*

Do you remember being taught the GALS examination approach whilst at medical school? **YES/NO**

Do you use the GALS approach when examining the musculoskeletal system in your current clinical practice? **ALWAYS / SOMETIMES / NEVER**

Do you feel that the GALS approach has improved your ability to detect significant musculoskeletal pathology in adult patients? **GREATLY IMPROVED / SLIGHTLY IMPROVED / NO EFFECT**

How would you rate your experience of the GALS approach? Please select your

preferred responses to the items listed below. *(5 point Likert scale: strongly agree, agree, neutral, disagree, strongly disagree)*

1. It is easy to remember
2. It comprehensively covers the full range of musculoskeletal examination expected of a non-specialist
3. It has sufficient detail in examination technique
4. It is sufficiently concise for routine use
5. It is sufficiently detailed to detect the vast majority of musculoskeletal pathologies

What do you like about the GALS approach? (optional) **Free text**

What do you dislike about the GALS approach? Can you suggest any ways in which this approach could be improved? (optional) **Free text**

REMS (Regional Examination of the Musculoskeletal System) Approach

When examining patients, do you distinguish between a general screening musculoskeletal examination versus detailed individual joint examination routines? **YES/NO**

At medical school, were you taught any specific examination routines for the following individual joints or joint groups? (please select as many as apply)

1. hands/wrists
2. elbows
3. shoulders
4. hips
5. knees
6. ankles/feet
7. spine

Are you aware of the REMS approach (Regional Examination of the Musculoskeletal System)? **YES/NO**

*If NO, then skip to next section*

Do you remember being taught the REMS approach whilst at medical school? **YES/NO**

Do you use the REMS approach when examining the musculoskeletal system in your current clinical practice? **ALWAYS / SOMETIMES / NEVER**

Do you feel the REMS approach has helped to improve your ability to detect pathology of specific joints? **GREATLY IMPROVED / SLIGHTLY IMPROVED / NO EFFECT**

How would you rate your experience of the REMS approach? Please select your

preferred responses to the items listed below. *(5 point Likert scale: strongly agree, agree, neutral, disagree, strongly disagree)*

1. The examination routines are easy to remember
2. They comprehensively cover the full range of musculoskeletal examination expected of a non-specialist
3. The examination routines are sufficiently detail
4. The examination routines are sufficiently concise for routine use
5. The examination routines are sufficiently detailed to detect the vast majority of musculoskeletal pathologies

What do you like about the REMS examination routines? (optional) **Free text**

What do you dislike about the REMS approach? Can you suggest any ways in which this approach could be improved? (optional) **Free text**

pGALS (Paediatric Gait-Arms-Legs-Spine Approach)

During your medical school training, were you taught how to perform a musculoskeletal examination of a school-aged child? **YES / NO / CAN’T REMEMBER**

Are you aware of the pGALS (paediatric Gait-Arms-Legs-Spine) approach to musculoskeletal examination in school-aged children?

**YES/NO**

*If NO, then skip to end of questionnaire*

Are you aware of the Arthritis Research UK educational video for pGALS examination?

**YES/NO**

*If YES*: Have you watched the Arthritis Research UK educational video for pGALS examination?

**YES/NO**

*If YES*: How have you viewed the Arthritis Research UK pGALS video? (select as many as apply)

1. DVD provided by Arthritis Research UK
2. Online video via Arthritis Research UK website
3. Online video via YouTube
4. Other (please specify below) **Free text**

Do you use the pGALS approach when examining the musculoskeletal system of school-aged children in your current clinical practice? **YES / NO / NOT APPLICABLE (my job never requires me to examine children)**

*If NO*: Do you use any alternative approaches to examining the musculoskeletal system of school-aged children? What approach do you use? **Free text**

*If* YES: Do you feel that the pGALS approach has improved your ability to detect significant musculoskeletal pathology in school-aged children? **GREATLY IMPROVED / SLIGHTLY IMPROVED / NO EFFECT**

How would you rate your experience of the pGALS approach? Please select your

preferred responses to the items listed below. *(5 point Likert scale: strongly agree, agree, neutral, disagree, strongly disagree)*

1. It is easy to remember
2. It comprehensively covers the full range of paediatric musculoskeletal examination expected of a non-specialist
3. It covers sufficient detail in examination technique
4. It is sufficiently concise for routine use
5. It has sufficient scope to detect the vast majority of significant joint abnormalities in paediatric musculoskeletal medicine

What do you like about the pGALS approach? (optional) **Free text**

What do you dislike about the pGALS approach? Can you suggest any ways in which this approach could be improved? (optional) **Free text**

End of questionnaire

*If eligible:*

Thank you for taking the time to complete the survey your responses will be collected and processed anonymously.

If you would like to be entered in the free prize draw, please enter your email address in the box below (optional). Please note that only doctors.org.uk/doctors.net.uk email addresses are accepted for entry to the free prize draw. **Free text**

*If not eligible:*

Unfortunately you fall outside of the target audience for this survey.

Thank you for your time and your participation is much appreciated.

If you would like to be entered in the free prize draw, please enter your email address in the box below (optional). Please note that only doctors.org.uk/doctors.net.uk email addresses are accepted for entry to the free prize draw. **Free text**
